# Rapid Invasion of *Spartina Alterniflora* in the Coastal Zone of Mainland China: Spatiotemporal Patterns and Human Prevention

**DOI:** 10.3390/s19102308

**Published:** 2019-05-19

**Authors:** Dehua Mao, Mingyue Liu, Zongming Wang, Lin Li, Weidong Man, Mingming Jia, Yuanzhi Zhang

**Affiliations:** 1 Key Laboratory of Wetland Ecology and Environment, Northeast Institute of Geography and Agroecology, Chinese Academy of Sciences, Changchun 130102, China; maodehua@neigae.ac.cn (D.M.); liumy917@ncst.edu.cn (M.L.); manwd@ncst.edu.cn (W.M.); 2 College of Mining Engineering, North China University of Science and Technology, Tangshan 063210, China; 3 Department of Earth Sciences, Indiana University-Purdue University, Indianapolis, IN 46202, USA; ll3@iupui.edu; 4 Center for Housing Innovations, The Chinese University of Hong Kong, Shatin, New Territories, Hong Kong 999077, China; 5 Key Lab of Lunar Science and Deep-exploration, National Astronomical Observatories, Chinese Academy of Sciences, Beijing 100101, China

**Keywords:** exotic species, spatiotemporal patterns, *S. alterniflora* invasion, CAS *S. alterniflora*, Landsat images

## Abstract

Given the extensive spread and ecological consequences of exotic *Spartina alterniflora* (*S. alterniflora*) over the coast of mainland China, monitoring its spatiotemporal invasion patterns is important for the sake of coastal ecosystem management and ecological security. In this study, Landsat series images from 1990 to 2015 were used to establish multi-temporal datasets for documenting the temporal dynamics of *S. alterniflora* invasion. Our observations revealed that *S. alterniflora* had a continuous expansion with the area increasing by 50,204 ha during the considered 25 years. The largest expansion was identified in Jiangsu Province during the period of 1990–2000, and in Zhejiang Province during the periods 2000–2010 and 2010–2015. Three noticeable hotspots for *S. alterniflora* invasion were Yancheng of Jiangsu, Chongming of Shanghai, and Ningbo of Zhejiang, and each had a net area increase larger than 5000 ha. Moreover, an obvious shrinkage of *S. alterniflora* was identified in three coastal cities including the city of Cangzhou of Hebei, Dongguan, and Jiangmen of Guangdong. *S. alterniflora* invaded mostly into mudflats (>93%) and shrank primarily due to aquaculture (55.5%). This study sheds light on the historical spatial patterns in *S. alterniflora* distribution and thus is helpful for understanding its invasion mechanism and invasive species management.

## 1. Introduction

Biological invasion has been one of the most prevalent ecological issues in the worldwide coastal wetlands [1,2,3]. *Spartina alterniflora* (*S. alterniflora*), a species native to the Atlantic coastal America, has invaded worldwide from southern England (50° N) to South Africa and New Zealand (40° S) and caused serious ecological consequences including altered traditional landscape, threatened biodiversity, and degraded ecosystem functioning [4]. In response to such consequences, sustainable coastal ecosystem management and ecological security requires comprehensive understanding of the spatial pattern and temporal changes of *S. alterniflora* invasion.

China’s coastal wetlands play critical roles in providing ecosystem services but are being dramatically modified by natural (i.e., sea level rise) and human interferences [5,6]. The coast of mainland China is 18,000 km long and serves as important habitats for large populations of migratory birds, fish, and other animals including crustaceans, mollusks, and amphibians [7]. Although a large number of nature reserves have been established to protect endangered species and coastal wetland ecosystems, wetland degradation and biodiversity loss were striking due to multiple driving forces and inefficient protection [8]. The rampant spread of exotic *S. alterniflora* should not be overlooked as one of these driving forces. *S. alterniflora* was first introduced into China in 1979 for ecological engineering, planted over multiple sites in 1980s, extensively spread in 1990s, and thus listed as one of the most severe invasive plants by the State Environmental Protection Administration of China in 2003 due to its various negative consequences, particularly changing the habitats of water birds [9]. Therefore, it is urgent to effectively delineate the invasion pattern and determine the temporal dynamics of *S. alterniflora* in the coasts of mainland China.

Numerous studies attempted to examine the invasive dynamics of *S. alterniflora* at the regional or local scale in China. Traditional ground surveys cannot easily provide a broad-scale understanding of the extent of *S. alterniflora* invasion or variation in colonization rates. Remote sensing is a feasible approach for understanding the changed vegetation pattern of wetlands [10,11,12] and thus is widely used in examining the expansion of *S. alterniflora* in China. Multiple-source remote sensing data were used to investigate the invasion of *S. alterniflora* in Jiuduansha shoals of Shanghai [13], Beihai City of Guangxi [14], Yueqing Bay of Zhejiang [15], and Zhangjiang Estuary of Fujian [16], and so on. Among which, Landsat images have been widely used due to moderate spatial resolution, broad coverage, and consistent availability since the 1980s [15,17].

Although previous studies revealed the distribution of *S. alterniflora* invasion across mainland China [18,19,20], little is known about the temporal pattern of *S. alterniflora* invasion at the scale of mainland China due to scarcity in multi-temporal data. Such information shortage has limited our understanding of the mechanism of *S. alterniflora* invasion and inhibited scientific decision-making. In a previously published paper [17], the up-to-date pattern of *S. alterniflora* invasion was delineated by applying the object-based image analysis (OBIA) and support vector machine (SVM) to Landsat 8 Operational Land Imager (OLI) images acquired in 2015. In this study, the 2015 *S. alterniflora* invasion dataset and change detection were applied to historical Landsat Thematic Mapper (TM) and Enhanced Thematic Mapper Plus (ETM+) images to create a long-term observation for documenting the dynamics of exotic *S. alterniflora* invasion in mainland China. In this paper, we aimed (1) to quantify the temporal changes of *S. alterniflora* area at decadal intervals from 1990 to 2015; (2) to examine the spatial variances in *S. alterniflora* invasion at scale of province and city; and (3) to investigate the conversions between *S. alterniflora* and other types of land cover and reveal artificial prevention of the spread of *S. alterniflora* in mainland China. The generated datasets and related analysis in this study are expected to help manage coastal zones and ensure ecological security.

## 2. Materials and Methods

### 2.1. Coastal Mainland China

**Figure 1:**
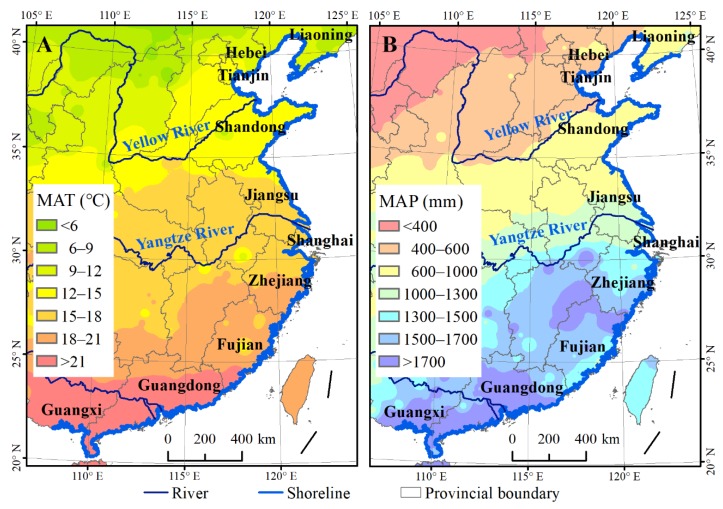
Geographic location and climatic gradients of the study area: (**A**), mean annual temperature (MAT); (**B**), mean annual precipitation (MAP).

The coastal zone of mainland China (Figure 1) spans 10 provinces (Guangxi, Guangdong, Fujian, Zhejiang, Shanghai, Jiangsu, Shandong, Tianjin, Hebei, and Liaoning), ranging from 20° N to 41° N in latitude and 108° E to 124° E in longitude. This region has a warm temperate climate in the north to a subtropical climate in the south, with the mean annual temperature (MAT) varying from 5 to 25 °C and the mean annual precipitation (MAP) from 400 to 1800 mm (Figure 1). The common intertidal plants include *Phragmites australis*, *Suaeda salsa*, *Tamarix chinensis*, *Scirpus mariquete*, *Cyperus malaccensis*, and mangrove forests such as *Aegiceras corniculatum, Avicennia marina, and Kandelia candel*. Exotic *S. alterniflora* is the most serious invasive plant, and has expanded to dominate over mangroves [17,21]. As the most developed region in China, the coastal region of mainland China has been significantly affected by notable human activities [22,23].

### 2.2. Satellite Data and Preprocessing

Landsat is recognized as the only data source for moderate resolution observations over a long time series. To examine the spatiotemporal patterns of *S. alterniflora* invasion, cloud-free images acquired from 1990 to 2015 by TM of Landsat 5, ETM+ of Landsat 7, and OLI of Landsat 8 were downloaded from the United States Geological Survey (USGS, https://glovis.usgs.gov/). Multi-temporal images in specific locations were selected to use so that the separation of *S. alterniflora* from other species was maximized based on the phenological stages and tidal level. Because tidal flooding reduces spectral reflectance and can add noise to the classification of *S. alterniflora*, images with low tidal heights were preferred, so that the *S. alterniflora* located in deep water could be easily identified. We used the China Network of Shipping Service (CNSS) website to determine the tidal height for each time and location (http://app.cnss.com.cn/tide_search.php). Moreover, we used the image acquisition time-stamp to select images collected during low tide conditions as much as possible. A total of 152 scenes of images including 36 scenes in 1990, 34 scenes in 2000, 39 scenes in 2010, and 43 scenes in 2015 were collected to cover the whole study area. All the images were processed using the Fast Line-of-sight Atmospheric Analysis of Hypercubes (FLAASH) model for atmospheric correction and geo-rectified using the Environment for Visualizing Images (ENVI) 5.0 image processing software package with reference to ground control points (GCPs) selected on 1:50,000 topographic maps [21]. For each image, at least 30 evenly distributed GCPs were selected, and the root mean squared error of the geometric rectification was less than 0.5 pixel (or 15 m). The World Geodetic System 1984 (WGS84), universal transverse Mercator (UTM) zones 49–51 N projection system were used for images to ensure the data consistency. Accurate geometric rectification, image registration, and a standard projection system allowed us to mosaic the classification results. To support later image classification, we also obtained advanced spaceborne thermal emission and reflection radiometer global digital elevation model (ASTER GDEM) data with a spatial resolution of 30 m from the USGS.

### 2.3. Field Data Collection

**Figure 2:**
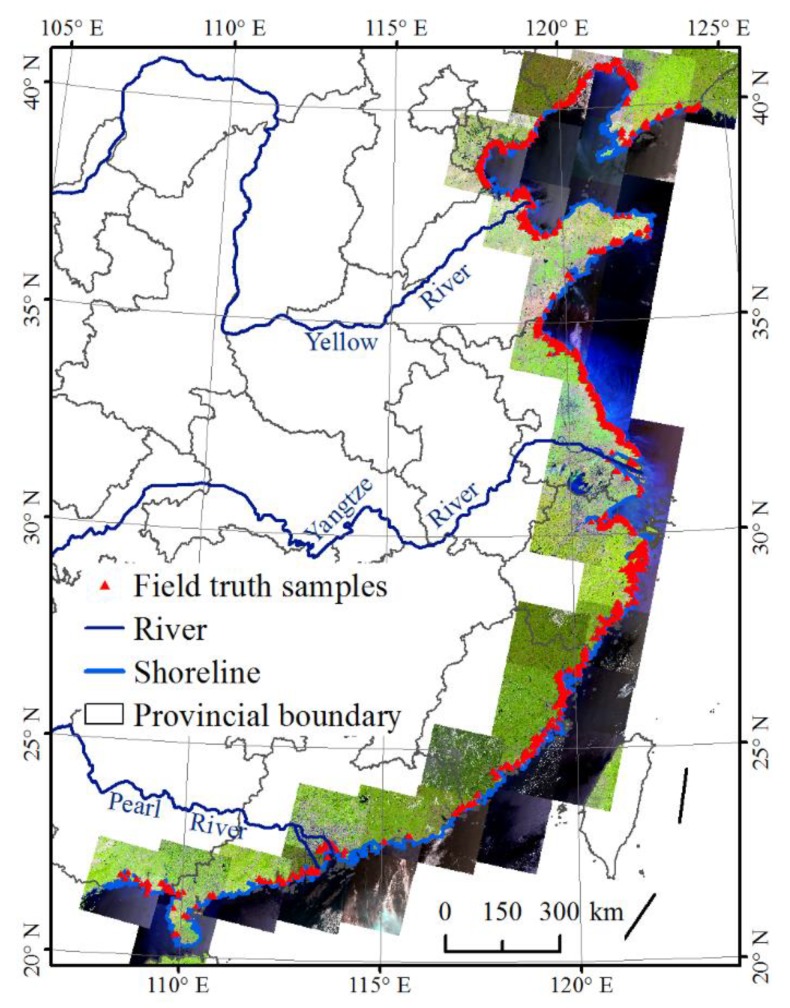
Landsat image coverage in 2015 and distribution of field ground-truth samples in 2015.

The field investigation was carried out between 2014 and 2016 to collect ground-truth points of *S. alterniflora* for supporting image classification and accuracy validation. The geographic locations of *S. alterniflora* were recorded using a hand-held geographic positioning system (GPS), and the photos at corresponding locations were taken using a digital camera for recording the ecological conditions at these locations. An unmanned aerial vehicle was used to examine the plant patches far away from the roads or coastal dams. Interviews with local residents were conducted to collect historical information about the distribution of *S. alterniflora*. A total of 1716 points covered by *S. alterniflora* and 9369 points around 2015 covered by other types of land covers were collected (Figure 2).

The ground-truth points (4473 for different wetland types) collected by previous studies in 2010 and 2015 for extracting coastal wetlands were also used to improve the classification in this study [21,24,25].

### 2.4. Methodology of Developing Multi-Temporal *S. Alterniflora* Datasets

*S. alterniflora* was first introduced in 1979, planted at multiple sites in 1980s, and spread rapidly from the 1990s in the coastal zones of China [9,18]. Therefore, this study has focused on mapping the distribution of *S. alterniflora* after 1990.

A detailed description on image classification for mapping *S. alterniflora* was given in [17]. Briefly, the method was composed of OBIA and SVM, two functions built in the eCognition Developer 8.64 software. Application of this method was composed of three critical steps. First, satellite images were segmented into homogeneous objects based on multiple parameters including scale, compactness, and shape. The fuzzy-based segmentation parameter (FbSP) optimizer was used to determine the optimal parameters for multi-resolution segmentation instead of employing the traditional trial method [17]. Second, SVM classifier was used to classify objects into specific categories by means of training samples obtained from field ground-truth points and a feature space composed of spectral, texture, and shape features. Third, manual editing was performed to inspect and modify initial classification results based on prior knowledge of in situ *S. alterniflora* and a great number of field ground-truth points. This process significantly increased the classification accuracy. These classification steps have been proven optimal for extraction of exotic *S. alterniflora* [15,17]. For avoiding duplicate classification due to overlay images, we used 33 grids to divide the study area for performing the classification. A buffer of 1 km was made for each grid to ensure the classification accuracy over the boundary area. Moreover, similar segmentation parameters were used for the adjacent grids to reduce the classification uncertainty from scale effects.

Following the combined method of OBIA and SVM [25], we used a change detection method to delineate changes in aerial cover of *S. alterniflora* from 2010–2015, 2000–2010, and 1990–2000. Images covering each grid in two years were simultaneously segmented into homogeneous objects in eCognition software. We could detect the differences between images in objects at the same location and thus were able to delineate changes in patches of *S. alterniflora* between different years [25]. These changed patches include two kinds of objects. For example, one kind of object was non-*S. alterniflora* in 2010 converted to *S. alterniflora* in 2015, while the other kind of object was *S. alterniflora* in 2010 converted to non-*S. alterniflora* in 2015. Visual interpretation was used to identify all the land cover classes of changed patches. We manually classified all the non-*S. alterniflora* objects into 10 land cover classes, including woodland, grassland, farmland, built-up land, barren land, marshes, mangroves, natural waterbodies, aquaculture ponds, and mudflats. For the manual classification process, we used the coastal land cover data and field samples acquired as part of our previous studies [21,25] as ground-truth data. Overlying the layers of *S. alterniflora* distribution in 2015 and changes of *S. alterniflora* between 2010 and 2015, the distribution of *S. alterniflora* in 2010 was generated in ArcGIS software. In sequence, the distribution datasets in 2000 and 1990 were generated using the same approach. In terms of the validation samples, overall accuracy of extracted *S. alterniflora* was 96%, while the kappa coefficient was 0.85. Producer accuracy was 91%, while the user accuracy was 86%. These results characterizing multi-temporal spatial distribution of *S. alterniflora* were finally integrated into the Chinese Academy of Sciences (CAS) *S. alterniflora* dataset [17].

### 2.5. Classification Result Analysis

The areal changes of *S. alterniflora* from 1990 to 2015 at decade scales were quantified and analyzed in terms of the geospatial pattern and rate of *S. alterniflora* invasion. Moreover, the conversion areas between *S. alterniflora* and other types of land cover were calculated and tabled with the ArcGIS software (ESRI version 9.3). For a better understanding on the contribution of various land cover types in expansion and shrinkage of *S. alterniflora*, the area ratio of the gain of *S. alterniflora* from each land cover type to the total expansion of *S. alterniflora* and the area ratio of the loss of *S. alterniflora* to each land cover type to the total shrinkage of *S. alterniflora* were respectively calculated and illustrated in bar graphs.

## 3. Results

### 3.1. The Dynamics of *S. Alterniflora* Invasion over the Whole Coastal Mainland China

Table 1 documents the areas of *S. alterniflora* in different years and their changes over different periods. Along the coast of mainland China, a continuous expansion of *S. alterniflora* was revealed with its area increased from 4376 ± 157 ha in 1990 to 25,648 ± 296 ha in 2000, 43,061 ± 401 ha in 2010, and 54,580 ± 649 ha in 2015. From 1990 to 2015, *S. alterniflora* has expanded by 50,204 ha, implying a mean net increase of 2008 ha·yr^−1^ or an annual change rate of 45.9%. *S. alterniflora* experienced the most rapid expansion in the period of 2010–2015 (2304 ha·yr^−1^), while the smallest expansion rate was found in the period of 2000–2010 (1741 ha·yr^−1^).

### 3.2. Pattern and Rate of *S. Alterniflora* Invasion

**Figure 3:**
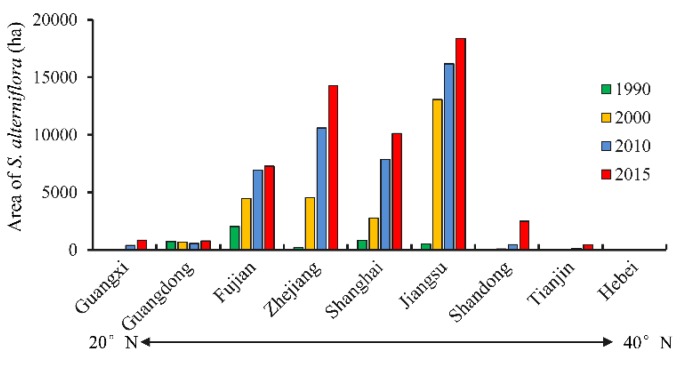
Spatial variation of *S. alterniflora* invasion.

According to Figure 3, inconsistent change patterns were evident over the coastal provinces during the investigated 25 years. The four central provinces, including Jiangsu, Shanghai, Zhejiang, and Fujian, experienced a dramatic expansion of *S. alterniflora* from 1990 to 2015, while the contribution from the north most (Tianjin, Hebei, and Shandong) and south most (Guangdong and Guangxi) provinces was limited.

The areal change of *S. alterniflora* over the different coastal provinces is shown in Table 2. During the first decade, 1990–2000, *S. alterniflora* in Jiangsu Province experienced the largest expansion with a net areal increase of 12,561 ha, while the smallest expansion was identified in Guangxi Province. However, *S. alterniflora* in three provinces (Tianjin, Hebei, and Guangdong) had a net areal decline. During the second decade, 2000–2010, the most dramatic area increase of *S. alterniflora* was observed in Zhejiang (6053 ha), while *S. alterniflora* decreased only in Guangdong Province (132 ha). Moreover, the area of *S. alterniflora* in all the coastal provinces increased from 2010 to 2015, of which Zhejiang had the largest expansion (3683 ha) again. For the whole study period of 1990–2015, Jiangsu Province had the largest expansion in *S. alterniflora* with an invasive rate of 715 ha·yr^−1^, followed by Zhejiang (563 ha·yr^−1^), while Hebei Province experienced the smallest net areal increase of only 1.5 ha.

**Figure 4:**
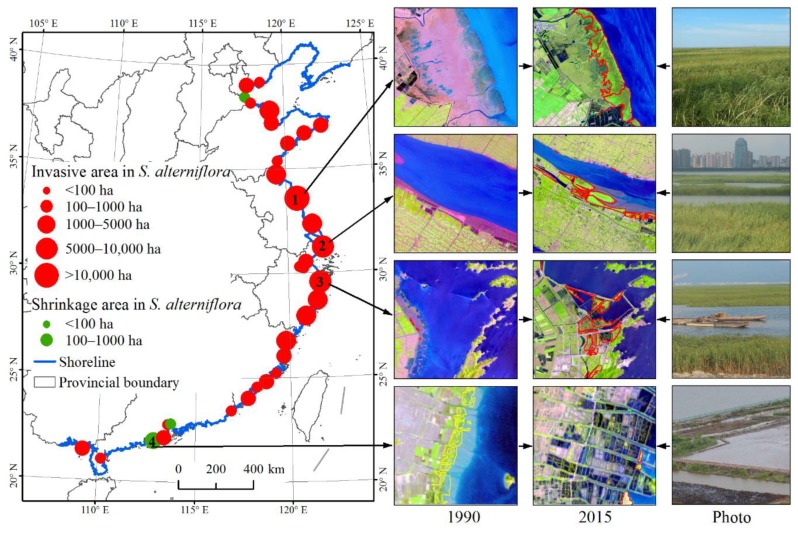
Areal changes of *S. alterniflora* between 1990 and 2015. The statistic of *S. alterniflora* area was obtained at the scale of city. Number 1 represents the location in Yancheng of Jiangsu; number 2 represents the location in Chongming of Shanghai; number 3 represents the location in Ningbo of Zhejiang; and number 4 represents the location in Jiangmen of Guangdong. Satellite images (composited from bands: red, green, and blue) provide examples for notable expansion and shrinkage of *S. alterniflora*; those corresponding photos were taken in 2015.

Figure 4 shows the areal change of *S. alterniflora* along the coast of mainland China between 1990 and 2015. The expansion of *S. alterniflora* was widely observed along the coast of mainland China, primarily in central provinces. Three noticeable hotspots of *S. alterniflora* invasion with a net area increase larger than 5000 ha were Yancheng of Jiangsu, Chongming of Shanghai, and Ningbo of Zhejiang (Figure 4). However, a distinct shrinkage of *S. alterniflora* was also identified over the coasts in three coastal cities including the Cangzhou of Hebei, Dongguan and Jiangmen of Guangdong. Among which, Jiangmen had the largest area decline (203 ha) during the observed 25 years due to land reclamation for the aquaculture development (Figure 4).

### 3.3. Conversions Between *S. Alterniflora* and Other Land Cover Types

**Figure 5:**
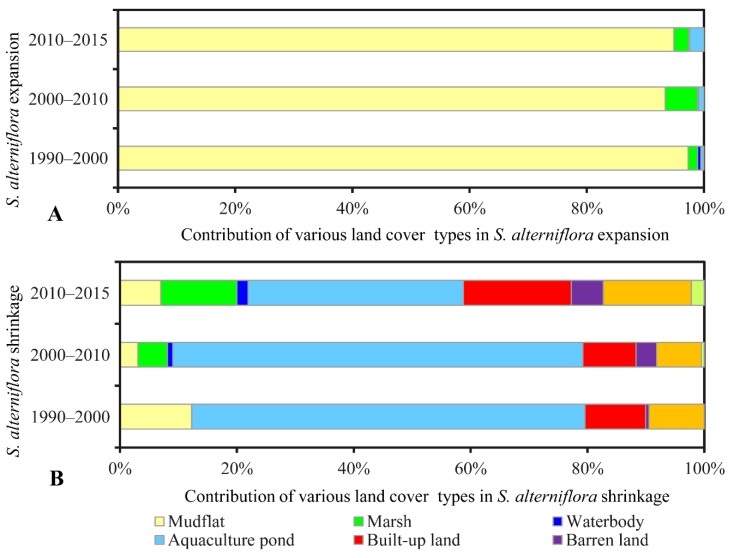
Contribution of various land cover types in *S. alterniflora* changes during different periods (contribution was calculated by area ratio): (**A**) expansion, (**B**) shrinkage.

The areas of other land cover types encroached by *S. alterniflora* are summarized in Table 3. During the investigated periods, the *S. alterniflora* encroached into other land cover types by a total area of 24,587 ha in period 1990–2000, 33,605 ha in period 2000–2010, and 26,357 ha in period 2010–2015. Specifically, *S. alterniflora* encroached mainly into mudflats (80,300 ha) with the contribution larger than 93% in each investigated period, which was significantly larger than that of any other land cover type (Figure 5A). In addition, *S. alterniflora* encroached into another largest two coastal wetland types, marsh and waterbody, with a total area of 2936 ha and 244 ha over the 25 years, respectively. Moreover, a total of 1047 ha of aquaculture pond was encroached by *S. alterniflora* at a continuously increasing pace during the investigated periods (Figure 5A).

In contrast to the extensive expansion of *S. alterniflora*, a large area of *S. alterniflora* at an increasing rate was converted into other land cover types (Table 4). The cumulative area of invasive *S. alterniflora* replaced by other land covers was 3314 ha, 16,192 ha, and 14,838 ha over the investigated three periods in order. Compared to the natural land covers, invasive *S. alterniflora* was mainly reclaimed for anthropogenic uses including aquaculture, infrastructure construction, and agriculture. As shown in Figure 5B, aquaculture ponds mainly contributed to the shrinkage of *S. alterniflora*, accounting for 67.3% of the shrinkage of *S. alterniflora* in 1990–2000, 70.2% in 2000–2010, and 36.9% in 2010–2015, and then were followed by built-up land and farmland. If we consider the conversion of *S. alterniflora* to aquaculture pond, built-up land, and farmland as the direct artificial elimination of *S. alterniflora*, human action in response to *S. alterniflora* invasion was evident with the converted *S. alterniflora* being 2887 ha during 1990–2000, 14,079 ha during 2000–2010, and 10,445 ha during 2010–2015.

## 4. Discussion

### 4.1. *S. Alterniflora* Invasion within China

Landsat provided a long time series of images and thus provided opportunity to generate the multi-temporal dataset of *S. alterniflora* invasion at scale of mainland China from 1990. In this study, the spatiotemporal patterns of *S. alterniflora* invasion observed from Landsat images noticeably revealed a serious invasion trend from 1990 to 2015 and a continuous expansion for most of the coasts. This result is consistent with the report of Zhang et al. [20]. Here, what should be highlighted is that *S. alterniflora* over the coast of mainland China experienced the most evident expansion in the period of 2010–2015 at a rate of 2304 ha·yr^−1^. Limited areas and sites artificially introduced *S. alterniflora* for ecological engineering of protecting seashores and capturing sediment, however, this exotic species was recognized to be the most serious invasive plant over the coastal zone in 2003 by the State Environmental Protection Administration of China. This study highlighted that the rampant extension of *S. alterniflora* was not yet effectively prevented. Previous studies revealed that the two top hotspot sites of *S. alterniflora* invasion were the coastal areas of Yancheng in Jiangsu and the Yangtze River Estuary in Shanghai [26,27,28]. Results from this study also indicate that these two sites experienced the largest area increase of *S. alterniflora* (Figure 4) during the investigated 25 years. Currently, Jiangsu Province has been determined to have the largest area and the highest rate of *S. alterniflora* invasion during 1990–2015 due to its extensive mudflats in the coastal zone, while expansion of *S. alterniflora* in Zhejiang Province were more evident due to the large areas of *S. alterniflora* in Jiangsu that were artificially converted to aquaculture ponds during the period of 2000–2015. The most rapid invasion of *S. alterniflora* in the four coastal provinces (Figure 3 and Table 2) necessitates the prevention of further invasion of *S. alterniflora* in other coastal provinces, particularly in Liaoning Province where *S. alterniflora* was not detected by our study, and Shandong Province where the area of mudflat is the largest among the coastal provinces [7]. Because *S. alterniflora* is highly adaptable, its range has expanded across the coast of mainland China and the Pacific coast of America [4,17]. Considering potentially high risks for *S. alterniflora* invasion into those coastal areas such as areas with a latitude from 20° N to 50° N where *S. alterniflora* has not been dominantly invasive, it is necessary to implement an effective mitigation strategy for addressing ecosystem health and regional security. Thus, our study provides important baseline information about *S. alterniflora* cover and expansion rates which can help inform monitoring and management programs aimed at reducing the range of this species in China.

### 4.2. Potential Driving Forces for the Spread of *S. Alterniflora* and Human Prevention in Mainland China

While our analytical methods were focused on mapping *S. alterniflora* expansion, a summary of the known driving forces and ecological effects of *S. alterniflora* invasion will help place these results in context. *S. alterniflora* invasion in mainland China primarily manifests as natural spreading. Climate factors such as mean annual air temperature and annual precipitation are expected to drive *S. alterniflora* spreading [29]. The climate in central provinces (Jiangsu, Shanghai, Zhejiang, and Fujian) was optimal for the ecological niche of *S. alterniflora* growth, but not in the north most coastal province, Liaoning, and a few areas in Hebei, Tianjin, and Shandong where lower temperature in spring may limit the seed germination or rhizomes proliferation of *S. alterniflora*. This implies that close attention should be paid to expansion of *S. alterniflora* toward these provinces as the climate warms.

**Figure 6:**
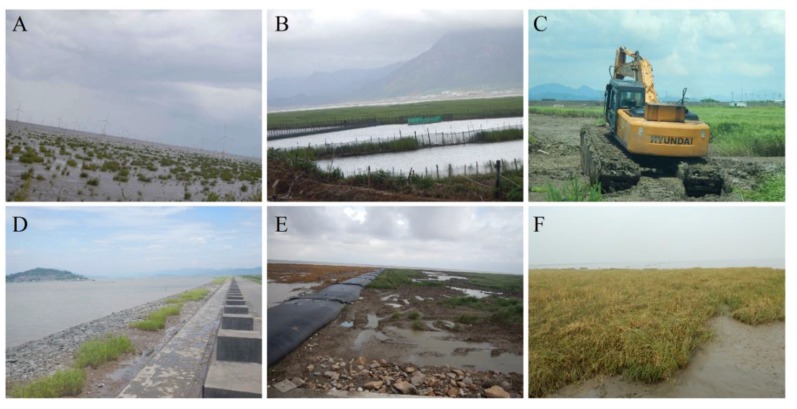
Photos documenting anthropogenic drivers for the expansion and shrinkage of *S. alterniflora*: (**A**) artificial planting, (**B**) aquaculture development, (**C**) agricultural cultivation, (**D**) dam and road construction, (**E**) herbicide treatment, and (**F**) spikes removal.

Anthropogenic activities play a secondary role in driving the distribution of *S. alterniflora* in mainland China. Artificial planting over multiple areas in the coastal provinces has promoted the spread of *S. alterniflora* (Figure 6A). As we have observed, invasive *S. alterniflora* encroached on extensive mudflats with an area of 80,300 ha during the considered 25 years (Table 3). However, conversions of *S. alterniflora* to other land cover types was mostly motivated by economic activities, and this was the one important factor that limited the spread of *S. alterniflora* in some areas. For example, more than 19,066 ha of *S. alterniflora* was converted to aquaculture ponds (Figure 6B), and 3787 ha reclaimed for grain production from 1990 to 2015 (Figure 6C). The infrastructure construction, including seaside dams, roads, and ports replaced 4558 ha of *S. alterniflora*, and blocked further spreading of *S. alterniflora* (Figure 6D). Meanwhile, ecological consequences of *S. alterniflora* invasion are increasingly evident, and human prevention measures have increased. For example, herbicide was used to put down the *S. alterniflora* in the Chongming Dongtan Ramsar wetland for protecting native habitats of water birds (Figure 6E). The spikes of *S. alterniflora* were removed from the coast of Tianjin for controlling the spread of *S. alterniflora* (Figure 6F). However, anthropogenic removal of *S. alterniflora* is inefficient, and thus natural succession combined manual intervention such as waterlogging should be developed to prevent *S. alterniflora* from expanding in the coastal wetland ecosystems.

### 4.3. Ecological Effects and Management Implications

**Figure 7:**
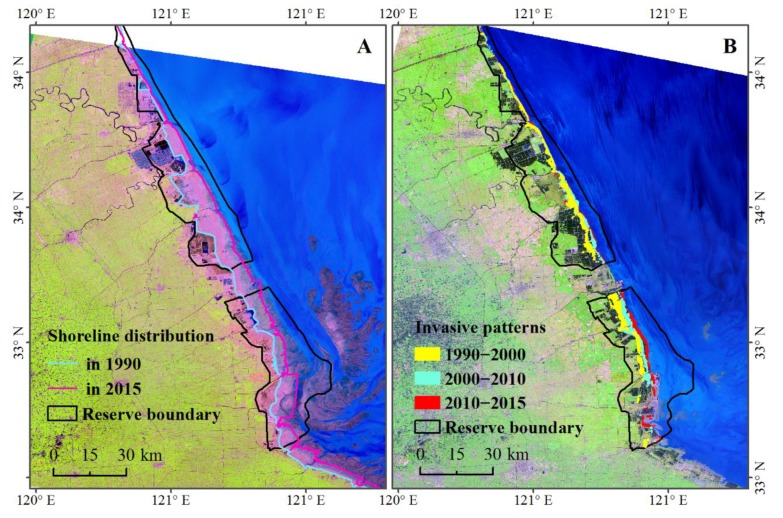
Change in shoreline (**A**) and invasive patterns of *S. alterniflora* (**B**) from 1990 to 2015 in Yancheng Ramsar site; the Landsat Thematic Mapper (TM) image (A) was obtained in 1990, while Landsat Operational Land Imager (OLI) image (B) was obtained in 2015.

Previous studies on the *S. alterniflora* invasion mostly reported the negative consequences [15,30,31]. Nonetheless, ecological effects of the *S. alterniflora* invasion are evaluated in positive and negative perspectives. On one hand, *S. alterniflora* greatly contributed to the seashore stabilization, dike protection, and tidal land reclamation in multiple sites of coastal provinces as was expected when *S. alterniflora* was artificially introduced. As shown in Figure 7A, the shoreline along the coast of Yancheng Ramsar site changed obviously from 1990 to 2015 as a result of introducing *S. alterniflora*. The planted *S. alterniflora* spread and formed an intercept barrier of sediments from sea water. The accumulation of sediments facilitated land reclamation for agricultural or aquaculture usage (Figure 7B). Meanwhile, the seaward land reclamation created potential space for *S. alterniflora* spreading resulting in a seaward *S. alterniflora* expansion trend (Figure 7B).

**Figure 8:**
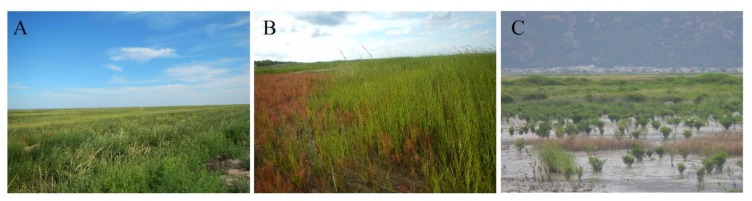
Photos indicating negative effects of invasive *S. alterniflora*: (**A**) affecting foraging habitats of water birds, (**B**) invasion upon native *Suaeda heteroptera* and (**C**) mangroves; all the photos were taken in 2016.

The most serious consequence caused by *S. alterniflora* invasion is its threat to native vegetation species. Dramatic encroachment of *S. alterniflora* into native ecosystems largely affected the habitat for water birds. For example, a large area of *Scirpus mariqueter* provided the primary food for *Anatidae* birds, but was replaced by exotic *S. alterniflora* in Chongming Island and Jiuduansha Shoals [32,33]. Consequently, many water birds had difficulty in obtaining food from the mudflats (Figure 8A). Additionally, some native plant communities were particularly vulnerable to *S. alterniflora* invasion, especially those dominated by *Suaeda heteroptera* (Figure 8B) and *Aegiceras corniculatum* mangroves (Figure 8C). The original functions and services of mangroves were thus altered.

Our previous study [17] revealed that the habitats for native species over the national nature reserves have been markedly invaded, suggesting an urgent need for prevention of *S. alterniflora* expansion and for the sake of coastal ecosystem conservation. Fortunately, during the period of 2010–2015, a large amount of *S. alterniflora* were replaced by native marsh (1856 ha) and mangrove (77 ha) in the context of artificial intervention that represented great potential for ecosystem rehabilitation. China’s central and local governments made great efforts to restore mangroves and eliminate *S. alterniflora* for coastal and water bird conservation [16,21]. As field investigations showed [21], mangrove plantings, including the exotic *Sonneratia apetala*, are effective for restraining the expansion of *S. alterniflora*. However, caution should be taken when a new exotic species is introduced. Both the positive and negative effects of *S. alterniflora* invasion should be considered for the coastal ecosystem conservation in China, and appropriate management decisions should be enacted after considering local coastal conditions.

## 5. Conclusions

Given its rapid expansion and notable ecological consequences, it is necessary to monitor the invasion of *S. alterniflora* for the sake of scientific ecosystem management and conservation. In this study, Landsat series images acquired from 1990 to 2015 were used to establish multi-temporal datasets for documenting the temporal changes of invasive *S. alterniflora*. This study provides the first detailed and long-term multi-temporal *S. alterniflora* distribution data for China. Our observations reveal the spatially distinctive variation of *S. alterniflora* invasion and a few hotspots at different scales. Although China’s government and local residents have made great efforts to prevent the expansion of *S. alterniflora* in specific regions, the rampant spread of *S. alterniflora* has not yet been effectively prevented. This *S. alterniflora* dataset sheds light on its historical and modern spatial patterns in *S. alterniflora* distribution and can help understand the mechanism of *S. alterniflora* invasion and develop effective ecosystem management. Given the risk of *S. alterniflora* invasion in China and other areas in the world, it is important to monitor *S. alterniflora* invasion with broad to fine scale spatial analyses that capture long term trends through modern methods such as artificial intelligence.

## Figures and Tables

**Table 1:** Areas and changes in *Spartina alterniflora* over the investigated 25 years.

Year	Area (ha)	Period	Change Area (ha)	Annual Change Rate
1990	4376 ± 157	1990–2000	21,273	48.6%
2000	25,648 ± 296	2000–2010	17,413	6.8%
2010	43,061 ± 401	2010–2015	11,519	5.3%
2015	54,580 ± 649	1990–2015	50,204	45.9%

**Table 2:** Changes in area of *S. alterniflora* over the different coastal provinces.

Province	Periods (ha)				Invasive Rate ha·yr^−1^
	1990–2000	2000–2010	2010–2015	1990–2015	
Hebei	−23	3	22	2	0.1
Tianjin	−13	112	305	405	16.2
Shandong	52	367	2037	2456	98.3
Jiangsu	12,561	3100	2201	17,862	714.5
Shanghai	1934	5102	2249	9285	371.4
Zhejiang	4330	6052	3683	14,065	562.6
Fujian	2427	2476	332	5236	209.4
Guangdong	−41	−132	224	51	2.0
Guangxi	45	332	466	843	33.7

**Table 3:** Encroached area of invasive *S. alterniflora* to various land cover types during the different study periods.

Land Cover Types	Encroached Area during Different Study Periods (ha)		
	1990–2000	2000–2010	2010–2015
Mudflat	23,917	31,379	25,004
Marsh	410	1855	671
Waterbody	131	67	45
Aquaculture pond	117	303	628
Barren land	12	1	7
Others	0	0	2
Total	24,587	33,605	26,357

**Table 4:** Converted areas of *S. alterniflora* to various land cover types during the different study periods.

Land Cover Types	Converted Area during Different Study Periods (ha)		
	1990–2000	2000–2010	2010–2015
Mudflat	406	494	1034
Marsh	0	729	1856
Mangrove	1	93	77
Waterbody	1	146	284
Aquaculture pond	2231	11,363	5473
Built-up land	343	1478	2737
Farmland	313	1237	2236
Barren land	19	571	815
Grassland	0	80	327
Total	3314	16,192	14,838
